# Cancer cell cooperation: tumor cells team up to scavenge nutrients to ensure proliferation despite starvation

**DOI:** 10.1038/s41392-025-02238-3

**Published:** 2025-05-12

**Authors:** Susanne Sebens, Lisa-Marie Philipp, Dieter Adam

**Affiliations:** 1 Institute for Experimental Cancer Research, Kiel University and University Hospital Schleswig-Holstein Campus Kiel, Kiel, Germany; 2 Institute of Immunology, Kiel University and University Hospital Schleswig-Holstein Campus Kiel, Kiel, Germany

**Keywords:** Cancer metabolism, Tumour heterogeneity

In a recent study published in *Nature*,^1^ Guzelsoy and colleagues demonstrate that tumor cells cooperate by sharing nutrients to ensure survival and tumor cell expansion even under nutrient scarcity. Moreover, their work identifies the aminopeptidase CNDP2 as an important molecule in this process and as a promising target for treating cancer.

So far, the traditional view has been that survival and expansion of individual tumor cells depend on successful competition for public goods such as nutrients and growth factors. However, it is well known from other ecological systems that cooperation of individual members is needed to cope with scarce conditions to ensure survival of the population. As nutrient scarcity is a characteristic feature of growing tumor ecosystems, Guzelsoy and colleagues postulated that, under these conditions, cancer cells team up to scavenge and share nutrients to promote cell growth.^1^ Referring to the Allee effect, (a positive effect of a larger population size on the fitness of an individual of a respective species^2^), the authors identified crucial cell collaborations within tumor populations, making them vulnerable for therapeutic targeting in order to eradicate the tumor. Habitat changes—which occur constantly in tumors—are an important factor impacting the Allee effect and favoring collaborative behavior.

Using different elegant experimental and analytical approaches in human and murine model systems, the authors identified a cooperative mechanism that relies on distinct tumor cells secreting the aminopeptidase CNDP2 which then hydrolyses extracellular oligopeptides. The resulting free amino acids in turn promote cell growth of the secreting as well as of neighboring tumor cells under amino acid-deprived conditions (Fig. 1). As CNDP2 loss resulted in diminished cell growth in glutamine-addicted cells under conditions of glutamine restriction, this mechanism of “cooperative scavenging of nutrients” seems to be vital for distinct tumors, e.g., tumors deficient of Kelch-like ECH-associated protein 1 (KEAP1).

**Fig. 1:**
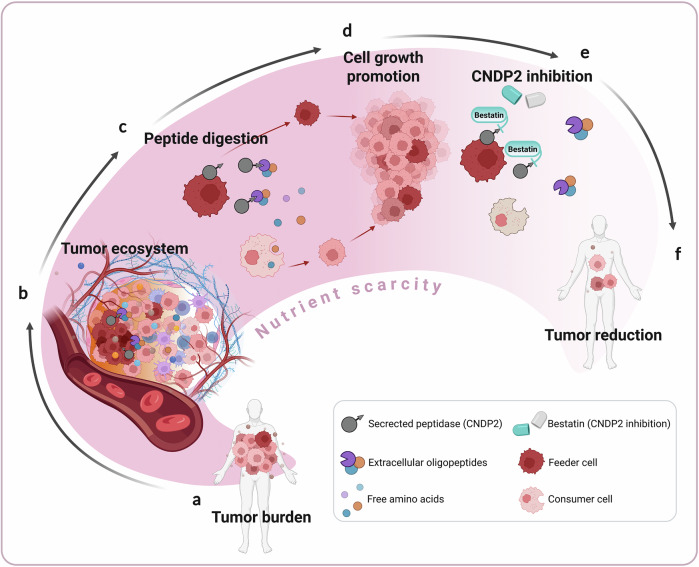
Cooperative nutrient scavenging ensures cancer cell proliferation despite starvation and its potential for therapeutic targeting. The illustration emphasizes how nutrient scavenging in starved cancer cells ensures survival and proliferation of the population. Panel (**a**) presents a cancer patient with a heterogenous tumor cell composition. This heterogenous tumor ecosystem (**b**) consists of different compartments, like blood vessels, immune and stromal cells as well as distinct cancer cell phenotypes. Both, the large population of densely packed patches of feeder cells (dark red) and the scattered, sparse patches of starving consumer cells (light red) coexist within the tumor. **c** Feeder cells have the ability to secrete the aminopeptidase CNDP2 which hydrolyses extracellular oligopeptides, resulting in free amino acids which in turn promote proliferation (**d**) of both feeder and consumer cells under amino acid-deprived conditions. **e** Loss or therapeutic inhibition of CNDP2, e.g., by administration of bestatin, results in a lack of free amino acids and therefore, prevents hydrolysis of extracellular oligopeptides and disrupts the nutrient scavenging of cancer cells. **f** This, in turn, may lead to decreased cancer cell proliferation and reduced tumor burden. This figure was created with BioRender.com

When cancer cells were starved of the amino acid glutamine, the cells needed to cooperate to survive and grow. In accordance with the Allee effect, these conditions benefited dense cell populations but not sparsely grown cell populations. By studying different tumor cell types, amino acids generated from extracellular oligopeptides by CNDP2 outside the cells were identified as key nutrient source that serve as public good for the entire tumor ecosystem (Fig. 1). Of note, this cooperative behavior was observed in different (malignant and benign) cell types pinpointing to a highly conserved mechanism. The fact that CNDP2 is a ubiquitously expressed non-specific aminopeptidase further suggests that CNDP2-mediated cooperative nutrient scavenging is not only used among tumor cell populations but rather represents a general cooperation mode, e.g., also between tumor and stroma/immune cells, driving tumor evolution and providing a fitness advantage to the entire tumor ecosystem.

Underscoring the key role of CNDP2 for cooperative nutrient scavenging, CNDP2 deletion or inhibition by treatment with the competitive aminopeptidase inhibitor bestatin prevented oligopeptide-dependent cell growth in every cell type tested and showed significant antitumoral effects in mouse models. Notably, the growth-inhibiting effects of bestatin could be rescued by adding free glutamine, excluding non-specific or toxic effects of the inhibitor. Regarding a clinical application, bestatin is already known for its low clinical toxicity and approved as adjuvant chemotherapy in Japan. Targeting CNDP2 may be particularly beneficial for lung cancer patients that frequently carry mutations in the *KEAP1* gene, as KEAP1 deficiency rendered cells more reliant on this cooperation. Furthermore, the therapeutic efficiency of CNDP2 targeting might be enhanced by combination with other strategies interfering with the immunosuppressive tumor ecosystem, e.g., immune checkpoint inhibitors.

Remarkably, long-term coculture competition and in vivo experiments revealed that non-cooperative (CNDP2 mutant) cells which only consume nutrients did not survive better but rather were driven to extinction by cooperative (CNDP2 wildtype) tumor cells. This suggests that dividing cooperative cells form a beneficial neighborhood of nutrient-producing cells whereas non-cooperators form a neighborhood without nutrient production and thus experience no benefit, also explaining why clonal patches are found in special niches in the tumor. These findings also support the view that the relative fitness of distinct tumor cell populations varies in dependence on spatial conditions, e.g., nutrient and oxygen supply or cellular composition.

In line with this concept, metabolic symbiosis has been already described between different tumor cell phenotypes as well as between tumor and stromal cells sharing metabolites thereby promoting tumor expansion and aggressiveness. Besides glucose or glutamine, tumor cells can also use lactate as energy source, which can be shuttled between neighboring cancer cells. This metabolic symbiosis can occur between hypoxic tumor cells, in which upregulation of HIF-1a promotes glycolysis and lactate production, and oxidative tumor cells, which take up the exported lactate for oxidative phosphorylation and thereby maximum energy gain. Similarly, stromal cells can also release lactate to fuel the metabolism of neighboring cancer cells.^3^ However, nutrient scarcity can also promote cellular competition, between tumor cells as well as between tumor and stroma/immune cells, the latter contributing to an immunosuppressive tumor ecosystem, thereby driving cancer evolution.^4^ Altogether, these findings suggest that both cellular competitions and cooperations may coexist within a tumor.

In order to survive and expand within such a dynamic tumor ecosystem tumor cells have to be able to switch their phenotypes and acquire different cell states. This phenotype switching is referred to as plasticity. Different phenotypes may exist within tumor cell populations which might either cooperate or compete within the tumor ecosystem, thereby providing an evolutionary advantage to the tumor, e.g., by promoting cell growth, invasion or therapy resistance.^5^ Elucidating the mechanisms underlying the origin and dynamics of those cancer cell phenotypes (and their plasticity) in cancer evolution is key to understanding tumor heterogeneity and how this contributes to tumor fitness pinpointing to vulnerabilities that can be therapeutically targeted. Thus, it can be speculated whether and how the described cooperative mechanism of nutrient scavenging is used by and dependent on distinct plastic phenotypes and how this is related to cellular functions beyond cell growth, e.g., migration and invasion. Adding to this heterogeneity, it has to be determined whether the CNDP2-dependent nutrient scavenging operates likewise in all tumors, tumor sites and tumor patients, respectively. Finally, considering the general glutamine abundance in the body, the heterogeneity of tumors and the cell’s metabolic plasticity, clinical translation of these findings requires further studies.

The study by Guzelsoy has contributed to a better understanding of intratumoral cooperation and has identified CNDP2 as a target structure with great therapeutic potential for tumor extinction. For translating this concept into the clinic, it will be important to further characterize the functional phenotypes of feeder and consumer cell clones. Another cornerstone will be to not only elucidate cooperations between tumor cells, but also bring stromal cells into this interplay which often are the dominant cell populations within the tumor ecosystem. As the tumor niche as well as tumor cell plasticity are important drivers of therapy resistance, it is also mandatory to better understand the role of cellular plasticity for intratumoral cooperations to ultimately identify the most vulnerable site(s) within this malignancy-promoting tumor collaboration.

## References

[1] Guzelsoy, G. et al. Cooperative nutrient scavenging is an evolutionary advantage in cancer. *Nature* **640**, 534–542 (2025). 39972131 10.1038/s41586-025-08588-w PMC11981941

[2] Courchamp, F. et al. (eds) Allee effects in ecology and conservation (Oxford Univ. Press, 2008).

[3] Doherty, J. R. & Cleveland, J. L. Targeting lactate metabolism for cancer therapeutics. *J. Clin. Invest.* **123**, 3685–3692 (2013). 23999443 10.1172/JCI69741 PMC3754272

[4] Yang, Y. et al. Glutamine metabolic competition drives immunosuppressive reprogramming of intratumour GPR109A^+^ myeloid cells to promote liver cancer progression. *Gut* **74**, 255–269 (2025). 38981667 10.1136/gutjnl-2024-332429

[5] Barkley, D., Rao, A., Pour, M., Franca, G. S. & Yanai, I. Cancer cell states and emergent properties of the dynamic tumor system. *Genome Res.* **31**, 1719–1727 (2021). 34599005 10.1101/gr.275308.121 PMC8494223

